# Assessment of infant and young child feeding practices and associated factors among less than two years' children in Leka Dullecha District, East Wollega, Ethiopia

**DOI:** 10.1002/hsr2.2119

**Published:** 2024-05-26

**Authors:** Fekadu Bulcha, Haile Bikila, Sidise Debelo, Chimdesa Tolera, Temesgen Tafesse, Ra'el Dessalegn, Desalegn Amenu

**Affiliations:** ^1^ Department of Public Health, Institute of Health Sciences Wollega University Nekemte Ethiopia; ^2^ Armauer Hansen Research Institute Addis Ababa Ethiopia; ^3^ East Wollega Zone Health Office, Health officer Nekemte Ethiopia; ^4^ Department of Biology, College of Natural and Computational Science Wollega University Nekemte Ethiopia

**Keywords:** breastfeeding, infant feeding practices, maternal practices, nutritional status

## Abstract

**Background and Aim:**

Leka Dullecha District, situated in East Wollega, Ethiopia, represents a region where infant and young child feeding practices play a crucial role in the health and well‐being of the population. Understanding the prevailing feeding practices among children under 2 years old is essential for devising effective interventions to improve child nutrition and reduce mortality rates. The context of this study involves examining various factors influencing infant and young child feeding (IYCF) practices, including socioeconomic, cultural, and environmental determinants. The main aim of the study was conducted to investigate the infant and young child feeding practice and associated factors among mothers of children 0–23 months in Leka Dullecha District, East Wollega, and Ethiopia.

**Method:**

A community‐based, cross‐sectional study design was carried out using 590 sample sizes. A stratified sampling method was used with simple random sampling technique. Bi‐variable and multivariable logistic regression models were used to identify factors associated with infant and young child feeding practices.

**Results:**

The overall prevalence of appropriate infant and young child feeding practice was estimated to 62.3%. According to this finding, age of child, place of delivery, and knowledge of mother were positively associated factors for inappropriate Infant and Young Child Feeding Practices.

**Conclusion:**

The overall infant and young child feeding practices in this study is not meeting the WHO guidelines for appropriate feeding practices. To achieve better feeding practices among 0–23 months aged children, intervention should focus on encouraging institution delivery and capacitating all health workers providing health education focusing on infant and young child feeding practices.

## 1 INTRODUCTION

Infant and young child feeding (IYFP) is defined as early initiation of breastfeeding (EIBF) within  1 h of birth, exclusive breastfeeding for 6 months and nutritionally adequate and safe complementary feeding starting from the age of 6 months with continued breastfeeding up to 2 years of age or beyond as recommended by United Nation international emergency fund (UNICEF) and World Health Organization (WHO).^1^ The first 2 years are the critical window for growth and development of children. If there is nutritionally deficit during this age period, it is difficult to redress later in life. The consequence of inappropriate IYCF during this critical time can lead children to falter physical growth, mental development, survival, and then reduce productivity and school performance too. Also it has negative implication on sustainable socioeconomic development and poverty reduction as a result of long term impact.^2^, ^3^ The overall, living standards in European or developed countries reflect a combination of economic prosperity, robust infrastructure, comprehensive social services, high‐quality healthcare and education, safety and security, environmental consciousness, and a range of cultural and recreational opportunities that contribute to a high quality of life for their residents. So, this disparity on adequate education and rapid economic growth as well as the availability of more facilitated health and nutritional infrastructure may lead poor and insufficient feeding practices in developing country, Ethiopia.

Globally, about 10.9 million deaths occur among less than five children annually due to the cases of malnutrition which accounted around 60.0%. The scaling up of breastfeeding to near universal levels can estimated to prevent 823,000 child deaths, which is corresponds to 13.8% of the deaths of children under 2 years of age.^4^, ^5^ Different strategies and guidelines are developed to realize the recommended standards of IYCF practices, which focuses on international code of marketing breast‐milk substitutes^6^ the innocent declaration,^7^ the millennium development goal, and global nutrition targets 2025, global strategy for IYCF^3^ guiding principles for complementary feeding of the breastfed child,^8^ are few of policies and strategies developed to strengthening IYCF practices.

Several studies have been conducted in several region of the country and the study revealed that, there is great challenges and problems which influencing infant feeding behaviors.^9^, ^10^ But practices of infant and young children feeding and associated factors has not been well studied in Ethiopia, particularly in the study area (Leka Dullecha). Most of the studies conducted in Ethiopia were not comprehensive and focused mainly on the breastfeeding aspects and not the dietary diversity and meal frequency. Hence there are significant gaps in our knowledge as to what amount of optimal IYCF and specific factors related to specific area with suboptimal child feeding at individual, house hold and community level. This study aimed to reveal the current practice of all IYCF components and associated factors which will be helpful for all sectors both government and NGO to have detail understand on magnitude of the child feeding problem and associated factors at all levels (from individuals to community level) in the area, this will enable stakeholders to clearly see the interventions need their effort to improve child nutrition practice and consider it during planning and play their role. So, this study was aimed to evaluate the infant and young child feeding practices and its associated factors among mothers of children 0–23 months in Leka Dellacha District, East Wollega Zone.

## 2 METHODS AND MATERIALS

### 2.1 The study area

**Figure 1:**
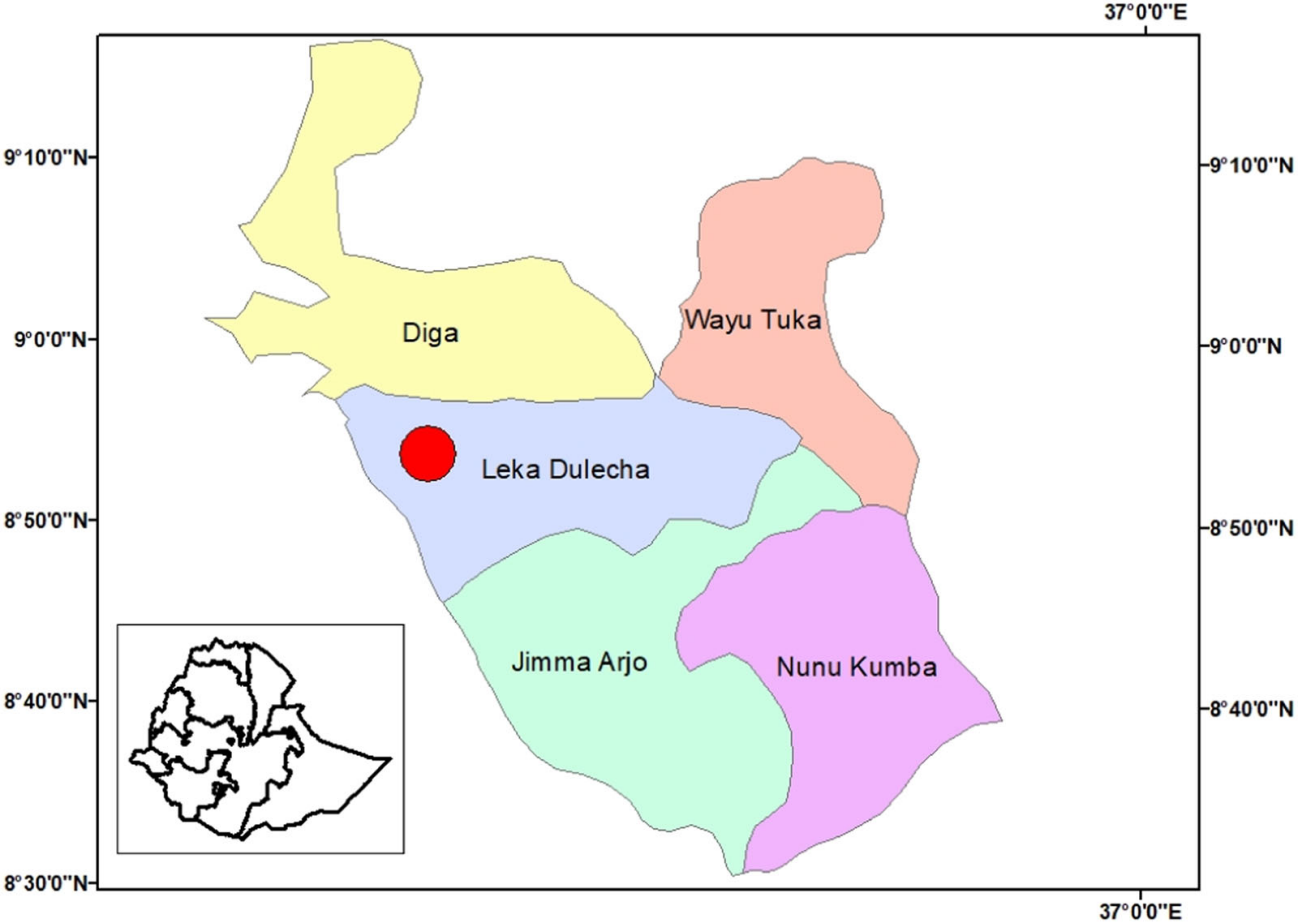
Map of the study area, East Wollega Zone (Leka Dulecha District).

The study was conducted in Leka Dulecha District, East Wollega Zone. It is 27 km far from Nekemte to the south and 358 km from Addis Ababa. The District is bordered by Diga, Jimma Arjo, Wayu Tuka and Chawaka Districts to the north, south, and east and to the west respectively (Figure 1). According to the District report, the total populations projected for 2022 fiscal year were 103,023 from this, 51,099 were men and 51,924 were women. This District has a total of 21,463 households and 2299 women in reproductive age group (15–49 years). Above 1 year's infants were 3314 and 0‐23 months children are accounted 5882 in the district, according to the information obtained from Leka Dulecha District Health office, the district has three public Health center, one NGO Health center which is not for profit, nine primary private clinics, two rural drug venders, and two drug store, Likewise one preparatory, two high schools, and 35 elementary School. The main economic activities are food crop, and livestock production. No exaggerated cash crops are produced, but some parts of the communities are produce khat and coffee. The most important crops produced are cereals, potatoes, vegetables and partially fruits.

### 2.2 The study design and period

Community based cross sectional study was conducted among mothers of children aged less than 2 years at time of the survey. While the study period was from May 2022 to September 2022.

### 2.3 Source of population

The source population for the study was all mothers who have child 0–23 months aged in Leka Dulecha District.

### 2.4 The study population

All mothers of children aged 0–23 months living in randomly selected kebeles of Leka Dulecha District.

### 2.5 Inclusion and exclusion criteria

All mothers of the child aged 0–23 months present during study period were include in this study, while, the mothers of children aged 0–23 months who are critically ill, and who has hearing impairment, as well as children having any illness 15 days before the survey were not included.

### 2.6 Sample size determination

The sample size required for the study was calculated using the formula to estimate a single population proportion.

$$n=\left(\frac{Z\alpha }{2}\right)2p\;\left(1-p\right)/d2$$

Where

n = Sample size

z = Level of confidence proportion with CI of 95% = 1.96

p = Proportion of appropriate infant and young child feeding practices 57.7% taken from study conducted in Kalu District, Northeast Ethiopia (31)

d = Margin of sampling error tolerated (0.05) 1.5 = design effect

n = 562 + 5% nonrespondent Total sample = **590**

Calculated by epi‐info with considering assumptions: power = 80%, two‐sided confidence level = 95%, COR of variables, 1.5 design effect and 5% nonrespondent rate

The sample size for objective 1 is 590; the maximum sample size among factors or objective 2 is 503, therefore, in this study the greater sample size 590 was used.

### 2.7 Sampling procedures

**Figure 2:**
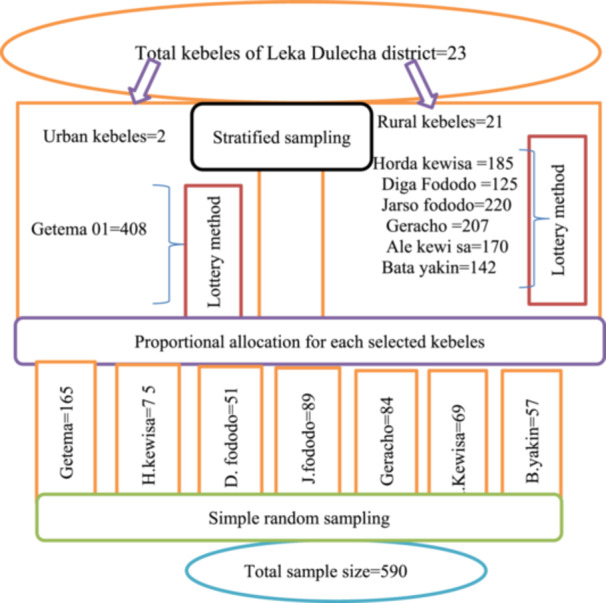
Schematic representation of the sampling procedure for the study on infant and young child feeding practice and associated factors among mothers of 0–23 months aged children in Leka Dulecha District, East Wollega, Ethiopia, 2022.

A stratified sampling technique was used. Initially the kebeles found in the District was stratified as urban and rural from total kebeles of Leka Dullecha District. Then one urban kebele and six rural kebele were selected by lottery method among total kebeles of the District. Lists of children age 0–23 months that is prepared by Health Extension Works from community health information system folder was used as a sampling frame (Figure 2). By taking all mothers of children 0–23 months age in Leka Dullecha District among seven selected kebeles, the proportional allocation of sample size was made for each selected kebeles by using the following statistical formula; ‐

ni=Ni∗n/N

Where ni = total sample size in kebele

Ni = total number of mothers of children 0–23 months aged in each selected kebele

$$n1=\frac{\text{Ni}\;\times \;n}{N}=\frac{408\times 590}{1458}=165$$

$$n2=\frac{N2\times n}{N}=\frac{185\times 590}{1458}=75$$

$$n3=\frac{N3\times n}{N}=\frac{125\times 590}{1458}=51$$

$$n4=\frac{N4\times n}{N}=\frac{220\times 590}{1458}=89$$

$$n5=\frac{N5\times n}{N}=\frac{207\times 590}{1458}=84$$

$$n6=\frac{N6\times n}{N}=\frac{170\times 590}{1458}=69$$

$$n7=\frac{N7\times n}{N}=\frac{142\times 590}{1458}=57$$

### 2.8 Data collection tools

Face to face interview was conducted by data collectors. Questionnaires were prepared and administered to the respondents. It was designed in a way to assess the experience of breast feeding, complementary feeding and the factors associated with the breast feeding and complementary feeding practice, in line with the WHO recommendations guidelines likes EIBF, EBF, timely initiation of CF, MMF, MDD, MAD and bottle feeding. For the sake of reliability most of the questions on the IYCF questionnaires were current status questions, based on recall of the very recent past (the previous day). In specific terms, this recall period starts from when the IYC awoke the previous day and extends throughout the day and night until its awaking on the morning of the interview day, for a total time period of approximately 24 h.

### 2.9 Data quality control

The quality of data was assured by properly designing and pretesting of the questionnaire, proper training was conducted for data collectors and supervisors on data collection procedures, proper categorization and coding of the questionnaire. Additionally, regular monitoring and supervision were implemented throughout the data collection process to ensure accuracy and consistency. Data validation checks were also performed to identify and correct any errors or inconsistencies in the collected data.

### 2.10 Operational definition

Appropriate

Inappropriate

Sufficient

Insufficient

### 2.11 Method of data analysis

After data Collection, data was checked manually for its completeness and consistency, then sorted, coded and entered to epidata version 3.1, and then it was processed by Statistical Packages for Social Science (SPSS) version 24. Further, data cleaning (editing, recording, checking for missing values, and outliers) was made after exported to SPSS. IYCF indicators were analyzed according to the formulas given in WHO IYCF guideline document.

### 2.12 Ethical considerations

Ethical clearance was obtained from Wollega University, Department of Public health ethical clearance committee. Official letter was written to the concerned bodies, and then the department head had approached. The respective bodies were asked to cooperate in the process of data collection after informing about the purpose of research project. Oral consent was obtained from the study participants after explaining the aim and purpose of the study. The participants were informed that they have a full right to participate or decline from participating in the study. Participants confidentiality was assured by excluding name as identification in the questionnaire.

## 3 RESULT

### 3.1 Socio‐demographic characteristics

Five hundred ninety (590) mothers who were sampled and had infants and young children between the ages of 0 and 24 months were successfully included in the study, with a response rate of 98.47%. The remaining informants did not respond. Table 1 show that the moms' manager was 26.22 years old, with a range of 18 to 43 years.

**Table 1:** Socio‐demographic characteristics of respondents in Leka Dulecha District, East Wollega, Oromia, Ethiopia, 2022 (*n* = 581).

Variables	Alternatives	Frequency	Percent
1. Marital status	Married	572	98.5
	Others	9	1.5
2. Religion	Protestant	341	58.7
	Orthodox	214	36.8
	Other	26	4.5
3. Maternal education	Illiterate	215	37
	Literate	366	63
4. Occupation	House wife	433	74.5
	Merchant	48	8.3
	Employee	55	9.5
	others	45	7.7
5. Resident place	Rural	416	71.6
	Urban	165	28.4
6. Monthly income	<= 999ETB	115	19.8
	1000–1999ETB	82	14.1
	2000–2999ETB	85	14.6
	>=3000ETB	299	51.5
7. Age	below 20	102	17.6
	20–29	322	55.4
	30–39	147	25.3
	above 40	10	1.7
8. Number of children	4 and below	500	86.1
	5 and above	81	13.9

### 3.2 Child characteristics

About half 313(53.9%) of children were male and 268(46.1%) of them were female. The age <6, 6–11 and 12–24 months were 233(40.1%), 149(25.6%) and 199(34.3%) respectively, with 9.5 mean age. Regarding to birth order, 25% of them were first birth while 75% born at sub‐ sequent order. Nearly half of children (52%) birth intervals between youngest child and his immediate older were greater than 2years and 23.8% of them were less than 2years (Table 2).

**Table 2:** Maternal and child characteristics in Leka Dulecha District, East Wollega (*n* = 581) 2022.

Items		Alternatives	Frequency	Percent
1) **Sex of child**		a) Male	313	53.9
		b) Female	268	46.1
2) **Age category of child**		a) <6 months	233	40.1
		b) 6–11 month	149	25.6
		c) 12–23 months	199	34.3
3) **Birth interval**		a) <2 years	138	23.8
		b) 1st birth	141	24.3
		c) >2 years	302	52
4) **ANC visit**		a) yes	539	92.8
		b) no	42	7.2
5) **Place of delivery**		a) home	150	25.8
		b) health facility	431	74.2
6) **ANC frequency**		a) <4	517	89
		b) >4	64	11
7) **PNC visit**		a) yes	326	56.1
		b) no	255	43.9

### 3.3 Maternal health services use characteristics

Majority of (92.8%) mothers attended ante natal care. From which, those who had ANC follow up, (89%) had less than four visits. During ANC follow up (48.4%) of mothers only received information about Infant and Young Child Feeding Practices among those who attend ANC visit. Most (74.2%) of mothers had gave birth at health facilities, among those, (70.6%) of them had spontaneous vaginal delivery. More than half (56.1%) of mothers had received PNC visit and out of all attendee (81.3%) of them had received counseling on IYCF practices during this PNC services (Table 2).

### 3.4 Breast feeding practices

As this finding showed that, majority of the mothers had showed good Brest feeding practices, hence, about 445 (76.6%) had initiated breastfeeding within 1 h of delivery, but the rest 136 (23.4%) of mothers had started breastfeeding after 1 h of birth as retrospective history of them were assessed. Regarding to the colostrum feeding, 519 (89.3%) of mothers had fed colostrum to their new‐ born babies, whereas 62 (10.7%) discarded colostrum due to mothers perceived as cause of abdominal cramp (46.8%) and dirty (32.3%).

Among 199 respondents of children age range from 12 to 23 months, 176 (88.4%) of respondents reported that they had continued breast feeding, while 23 (11.6%) of them had stopped. Regarding exclusive breastfeeding, from 198 mothers of 0–5 complete months, 156 (78.8%) of infants were exclusively breastfed in the last 24 h of the survey. This finding show that from all 581 respondents (26.2%) of them did practices feeding their child with bottle, whereas (73.8%) of them did not in the last 24 h of the survey (Table 3).

**Table 3:** Prevalence of IYCF practice by using WHO‐recommended indicators in Leka Dulecha District, East Wollega, Oromia, Ethiopia, 2022.

Items	Alternatives	Frequency	Percent
1) Early Initiation of BF, 0–23 months	1) within 1 h	445	76.6
	2) after 1 h	136	23.4
2) Prelactete fed 0–23 months	1) yes	44	7.6
	2) no	537	92.4
3) colostrum fed 0–23 months	1) yes	519	89.3
	2) no	62	10.7
4) EBF 0‐5complete months	1) Yes	156	78.8
	2) No	42	21.2
5) Continuing 12–23 complete months	1) yes	176	88.4
	2) no	23	11.6
6) Bottle fed 0–23 months	1) yes	152	26.2
	2) no	429	73.8
7) Timely initiation, 6–8 complete months	1) yes	52	46
	2) no	61	54
8) Meal frequency 6–23 complete months	1) 3 and above	316	82.5
	2) under 3	67	17.5
9) Diversity diet 6–23 complete months	1) 5 and above	73	19.1
	2) under 5	310	80.9
10) Acceptable diet 6–23 complete months	1) acceptable diet	68	17.8
	2) not acceptable	315	82.2
11) Ever breast feed 0–23 months	1) yes	581	100
	2) no	0	0
12) Attitude of respondent	1) negative attitude	173	29.8
	2) positive attitude	408	70.2
13) Information about IYCFP	1) yes	401	69
	2) no	180	31

### 3.5 Complementary feeding practices

Among children age 6–8 complete months, 52 (46%) started solid, semi‐solid and soft foods when they were 6–8 months' age. From total of 383 children aged 6–23 months, only 73 (19.1%) of children met the requirements for minimum dietary diversity (received five or more food groups from eight food groups including breast milk within 24 h preceding the survey as per the recommendations) and 316 (82.5%) of children met minimum meal frequency per day preceding 24 h of survey. The proportion of children age 6–23 months who met minimum acceptable diet composite of minimum dietary diversity and minimum meal frequency, was 68 (17.8%) and 315 (82.2%) didn't met requirement (Table 3).

### 3.6 Knowledge and attitude of mother's on IYCFP

Out of the total 581, about 281 (48.4%) of the respondents had sufficient knowledge of IYCFPs knowledge. Similarly, out of 581 respondents 408 (70.2%) had positive attitude and while the rest, 173 (29.8%) had negative attitude towards IYCFP (Table 3).

### 3.7 Infant and young child feeding practice status

In this findings, about 362 (62.3%) mothers were capable to feeding their children in appropriately while the rest, 219 (37.7%) of them were un able to give sufficient feeding services for their children's (Table 3).

### 3.8 Factors associated with infant and young child feeding practice

According to this finding, at the first 1 year of their children, mother practices better feeding than at older year. As child age being between 6 and 11 months 2.7 times more likely to have appropriate IYCF practice than those children age 12‐23 months (AOR = 2.7; 95% CI: (1.645, 4.615)). This finding again revealed, mothers who gave birth at health facility were 2.6 times more likely to have appropriate IYCF practice than mothers who gave birth at home (AOR = 2.6; 95% CI: (1.694, 4.007)). Mothers who had sufficient knowledge about IYCFPs were 2.6 times more likely to practice appropriate IYCF when compared with those who had insufficient knowledge about IYCFPs (AOR = 2.6; 95%CI; (1.782, 3.895)) (Table 4).

**Table 4:** Bi‐variable logistic regression analysis output in Leka Dulecha District, East Wollega, Oromia, Ethiopia, 2022 (*n* = 581).

Items		IYCFP status		COR (95% CI)	P‐value
		Appropriate N (%)	Inappropriate N (%)		
Knowledge of the respondents	Insufficient	158(62.7)	142(47.3)	1	
	Sufficient	204(72.6)	77(27.4)	2.38(1.684,3.367)	0.001*
Age category of child	<6	144(61.8)	89(38.2)	1.3(0.891,1.923)	0.170
	6‐11	108(72.5)	41(27.5)	2.1(1.352,3.360)	0.001*
	12‐23	110(55.3)	89(44.7)	1	
Birth interval	1st birth	74(53.6)	64(46.4)	1	
	≥2 years	90(63.8)	51(36.2)	1.5(0.945,2.465)	0.084
	<2 years	198(65.6)	104(34.4)	1.65(1.093,2482)	0.017
Age of mother	below 20	64(62.7)	38(37.3)	3.9(0.959,16.109)	0.057
	20‐29	202(62.7)	120(37.3)	3.9(0.997,15.476)	0.051*
	30‐39	93(63.3)	54(36.7)	4(0.997,16.190)	0.050*
	above 40	3(30)	7(70)	1	
Maternal education	illiterate	123(57.2)	92(42.8)	1	
	literate	239(65.3)	127(34.7)	1.41(0.996, 1.988)	0.052*
Education of father	illiterate	66(57.4)	49(42.6)	1	
	literate	296(63.5)	170(36.5)	1.29(0.854, 1.957)	0.225
Resident place	rural	244(58.7)	172(41.3)	1	
	urban	118(71.5)	47(28.5)	1.76(1.198,2.615)	0.004*
Number of children	≤4	320(64)	180(36)	1.65(1.029,2.648)	0.038
	≥5	42(51.9)	39(48.1)	1	
Sex of child	Male	187(59.7)	126(40.3)	0.78(0.562,1.106)	0.169
	Female	175(65.3)	93(34.7)	1	
Place of delivery	Home	65(43.3)	85(56.7)	1	
	Health facility	297(68.9)	134(31.1)	2.89(1.978,4.247)	0.001*

Significant at *p* value < 0.05; Reference Category = 1.

* =significant variable.

## 4 DISCUSSION

In this study, the prevalence of appropriate IYCF practice was 62.3% which was consistent with study conducted in Dangila district, North West of Ethiopia from which 62.5% was reported (83), also nearly similar with study conducted in Debrelibanos district 65.8%^11^ and Kalu District 57.7%^12^ in other hand it was higher than finding in Shashemene 32.1%^13^ in Affar, Assayita 9.2%.^14^ The deference might be due to the study setting that in current study consumption of iron‐rich or iron‐fortified foods was excluded while conducted in Affar asayita, and the finding from shashemene was emphasize only on seven indicators whereas eight in this study. In addition, time gap between study periods may be other reason for this difference.

In this study early initiation of breast feeding within 1 h of delivery was encountered 445(76.6%). This finding is similar finding in Kalu District, Northeast Ethiopia,^12^ but higher than findings in India,^14^ Afar^15^ and shashemene,^13^ this disparity might be due to high prevalence of mothers had information about IYCF practices and better in giving birth at health facility at study area.

In Ethiopia breastfeeding is universal, as some studies indicated, the prevalence of every breastfed is 95.6% in Asella and 99.3% in Shashemene.^9^, ^16^ Similarly in this study all respondent 581(100%) had reported as ever breastfeed their child and generally, the variation might be due to socioeconomic difference and breast feeding is normally known in the study area.

As recent study indicated the exclusive breastfeeding for children 0–5 complete months was 156(78.8%), it was similar with result obtained in Dire^17^ but lower than study finding in Bahir Dar,^18^ however it was higher than MDHS 2019 of Ethiopia, and Somaliland,^19^ this higher result may be due to high percent 74.2% of mothers gave birth at health facility and 70.1% of them had received information about IYCF practices in this study area. The present study revealed bottle feeding as 152(26.2%), which is consistence with study result in Affar Asayit,^15^ Bahir Dar city^18^ and far higher than finding in Pakistan (12%) and Ethiopian MDHS (9%) for children less than 6 months,^20^, ^21^ this discrepancy might be due to different age categories of children in capturing for study.

In current study the prevalence of continued breast feeding at age of 12–23 months children was 176(88.4%). The result shows nearly similar with finding in Debre‐libanos north showa oromia and in Bahir Dar. This was higher than study conducted in India^22^ for same age group, and the higher disparity was revealed which might be due to fact that continuing breast feeding till 2 years was becoming as a norm of this district societies.

In this study the prevalence of TICF among 6‐8 months aged children was 52(46%). It was higher than findings from Bangladesh, Nigeria, and India,^23^, ^24^, ^25^ this difference might be due to socioeconomic disparity. In other hand it was lower than study results in some parts of Ethiopia, like; in Kalu District northeast Ethiopia^18^, ^19^, ^26^ in Afar, North shoa and shashemenne respectively, this difference might be due to poor knowledge about IYCFP, as it was reported by less than half in current study and due to the study setup that 6‐8 complet months age children were assessed for last 24 h before survey in this study while retrospective history asked in other studies.

In present study 316(82.5%) of 6–23 months old children have been given the minimum meal frequency. This finding is line with finding in shashemene^13^ Debrelibanon district north shoa Oromia^11^ and nearly consistent with Kolkata India^22^ and Kenya.^27^ However it is higher than study result from Asella town,^28^ in Slum areas of Bahir Dar City,^18^ and in Jimma Zone.^29^

The prevalence of dietary diversity was only 73(19.1%) among 6–23 months aged children, who received five and above food categories out of 8 as recommended by WHO. Nearly all the children aged 6–23 months old consumed foods made from grains, roots and tubers beside breast milk mainly in form of porridge and ‟injera.” Women who gave flesh food to their child was almost nil, whereas eggs, Vitamin A rich fruits and vegetable, other fruits and vegetables were given by very few respondents. This is similar to studies in rural Damot sore district, Southern Ethiopia,^30^ and in Dangila Town, Northwest Ethiopia.^31^ This disparity of dietary diversity result might be due to socio economic factors and low knowledge of mothers about IYCFP among the current study area. In other hand this finding was higher than study from rural population of northwest Ethiopia^30^ and Bahir Dar Amhara.^32^ This high result might be due to awareness of mothers on the importance of diversifying diet in feeding children.

According to WHO definition, a child who met both the MDD and MMF are categorized as adequate minimum acceptable diet.^29^ The proportion of children who received the mini‐ mum acceptable diet was 68(17.8%), from Sheno town Oromia.^33^ In current study similar to study reported from Gorche district, Sidama Zone Southern Ethiopia,^34^ the most children failed to satisfy the MAD requirement largely due to suboptimal dietary diversity. According to this study, as age of child increase practicing appropriate IYCF was decrease. Child age being between 6 and 11 months was 2.7 times more likely to have appropriate IYCF practice than those children age 12‐23 months (AOR = 2.7; 95% CI: (1.645, 4.615)). This finding was coincide with national representative data of Ethiopian in 2021.^35^

This finding revealed that mothers who gave birth at health facility were 2.6 times more likely to have appropriate IYCF practice than mothers who gave birth at home (AOR = 2.6; 95% CI: (1.694, 4.007)). It was line with study reported from Assayita Affar,^36^ (AOR = 2.55, 95% CI (1.32, 4.93)), Kalu district^12^ (AOR = 1.977; 95% Cl (1.101, 3.552)), and slum of Bahir Dar city (AOR 2.4; 95% CI 1.1, 7.3)^32^ also with study in Asella town (AOR = 1.77(1.02, 3.06)).^16^ In this study mothers who had sufficient knowledge about IYCFPs were 2.6 times more likely to practice appropriate IYCF when compared with those who had insufficient knowledge about IYCFPs (AOR = 2.6; 95%CI; (1.782, 3.895)). This is supported with study conducted in Dangila Districty (AOR = 5.061, 95% CI: 2.465, 10.389),^37^ and study conducted in Debrelibanos(AOR = 2.82, 95% CI: (1.27, 26.26)) (Id et al. 2021). But in similar setup of study con‐ ducted in shashemenne and Kalu District,^13^, ^38^ it was found as nonsignificant.

The age of children being less than 12 months, children from mothers gave birth at health facilities, and mothers having knowledge to ward IYCFP were positively associated with appropriate infant and young child feeding practices. All concerned bodies should focus on health information communication on infant and young child feeding practices with giving due attention for indicators those recommended by WHO.

## 5 LIMITATION AND FURTHER STUDY

Collecting accurate data on feeding practices relies heavily on the respondents**'** ability to recall and report information, which can be influenced by factors like social desirability bias or memory lapses. In addition, the study focus on specific factors associated with feeding practices, potentially overlooking other important determinants such as cultural beliefs, access to healthcare, and maternal employment status. Conducting a longitudinal study could provide insights into how feeding practices evolve over time and their long‐term impact on child health and development.

Complement quantitative data with qualitative research to understand the underlying reasons behind certain feeding practices and the cultural context influencing them. Implement and evaluate interventions aimed at improving feeding practices, such as educational programs for caregivers or community‐based support systems. Nutritional Assessment: Include comprehensive nutritional assessments alongside feeding practices to understand the overall nutritional status of children in the region.

## AUTHOR CONTRIBUTIONS

**Fekadu Bulcha, Haile Bikila, Sidise Debelo** participating on Data collection, field work and Data conceptualization, in addition, they are participating 1st, 2nd and 3rd author. **Chimdesa Tolera, Desalegn Amenu** and **Temesgen Tafesse** were participating on data analysis, edition and conceptualization; furthermore, **Desalegn Amenu** is the corresponding author. Copy editing, translation, paraphrasing and finally edition was supervised and processed by **Desalegn Amenu**.

## CONFLICT OF INTEREST STATEMENT

The authors declare no conflict of interest.

## DECLARATION OF TRANSPARENT STATEMENT

We, the researchers involved in the assessment of Infant and Young Child Feeding Practices and Associated Factors among Children under 2 Years Old in Leka Dullecha District, East Wollega, Ethiopia, hereby declare our commitment to transparency in all aspects of our study. By upholding these principles of transparency, we aim to promote integrity, accountability, and trustworthiness in our research endeavors and contribute to the advancement of knowledge in the field of Infant and Young Child Feeding Practices.

## Data Availability

Data sharing not applicable to this article as no datasets were generated or analysed during the current study. The data used and analyzed during the current study are available within the manuscript.
